# Complement Fixation Test for Specific Antibody Detection against Bovine Brucellosis in Selected Peasant Association of Guto Gida District, East Wollega Zone, Oromia, Ethiopia

**DOI:** 10.1155/2021/6668167

**Published:** 2021-06-23

**Authors:** Gudeta Ararsa, Eyob Hirpa, Morka Amante

**Affiliations:** Wollega University, School of Veterinary Medicine, Department of Veterinary Science and Laboratory Technology, P.O. Box 395, Nekemte, Ethiopia

## Abstract

Brucellosis is one of the major zoonotic diseases yet prevalent in Ethiopia. It is contagious and has harmful effects on free animal movement and export. A cross-sectional study was conducted from November 2016 to April 2017 in the Loko and Uke peasant association of Guto Gida District. The goal of this study is to determine the seroprevalence and associated risk factors of bovine brucellosis. The questionnaire survey was conducted on 200 respondents to collect the livestock owner's perception of this disease. Eighty (80) blood samples were collected from grazing cattle above six months of age. Serum was tested by complement fixation test (CFT) for *Brucella* antibody detection. Pearson chi-square is used to assess the relation of independent variables such as sex, site, and age with seroprevalence in a 95% confidence interval if *P* value is less than 0.05, recoded as significant. Seroprevalence of bovine brucellosis in the present study is 22.5%. Bovine brucellosis prevalence recorded in Uke (28.6%) was higher than that in Loko (21.2%). Again bovine brucellosis is higher in males (30.4%) than females (19.2%). There is prevalence variation among site, sex, and age which is statistically insignificant (*P* value>0.05). Survey findings revealed that 52% (104/200) of respondents did not know the causes of abortion, while 48% (96/200) of them confirmed abortion in their herd and 64% (124/200) of respondents removed retained fetal membrane by themselves. Brucellosis is a contagious reproductive disease of cattle with zoonotic implications and needs to design and implement control measures aiming at preventing further spread of the disease.

## 1. Introduction

*Brucella* is a Gram-negative and facultative intracellular pathogen. It may affect a wide range of mammals including humans, cattle, sheep, goats, pigs, rodents, and marine mammals [1]. Brucellosis is an extremely contagious bacterial disease of zoonotic importance responsible for significant reproductive losses in animals.

Bovine brucellosis caused by *B. abortus* biovars is the most important disease in many countries around the world because of its economic importance [2, 3]. The infection is typically a disease of sexually mature animals, which merely affects the reproductive organs of infected animals [4]. It is associated with abortion at the first gestation (“abortion storm” in naïve heifers) and mainly caused by biovars biotype-1 of *Brucella abortus* [5, 6].

*Brucella* enters the body through the digestive tract, mucosal layers, intact skin, direct or indirect contact with excretion of the organisms in uterine discharge, following abortion, and ingestion of colostrums and milk of infected animals [7–9]. After infection, it may spread through blood and the lymphatic system to another organ where it infects the tissues. It causes reproductive failures (disorders) such as metritis, abortion in the last trimester or birth of an unthrifty newborn in the female, orchitis, and epididymitis with frequent sterility in male animals [10–14].

The prevalence and incidence of bovine brucellosis differ between herds, areas, management, and reproductive system in many countries [11]. The clinical disease is still common in the Middle East, Asia, Africa, South and Central America, Mediterranean Basin, and Caribbean regions. Eradication of *Brucella abortus* from the domesticated herd was reported to be nearly complete in the United States [15]. The disease has been reported to occur in most countries in Africa including the sub-Saharan region [16].

In the last two decades, several serological surveys have shown that bovine brucellosis is an endemic and widespread disease in Ethiopia [17], for instance, prevalence of 18.4% around Addis Ababa [18], 11.6% in Sidama region [19], 11.2% in East Showa zone [20], 7.61% in Arsi region [21], 4.9% in Tigray region [22], 4.2% in Southeast Ethiopia [23], 2.9% in Central Oromia [24], 2.4% in Jimma zone [25], 1.97% in west Ethiopia, East Wollega zone, in Guto Gida district, from the animals that come to the clinic for the treatment [26], and 1.11% in Addis Ababa and Sululta abattoir [27].

In Ethiopia, different researches on seroprevalence of bovine brucellosis were performed at different times, which focused on the area where the facilities are available and a questionnaire survey does not support their findings. Therefore, the present study was conducted to provide information on the prevalence of bovine brucellosis based on the questionnaire survey and serological study technique.

### 1.1. Objectives

To estimate the seroprevalence of bovine brucellosis in the study area. To assess risk factors for bovine brucellosis in the study area. To assess knowledge, attitude, and practice of owner of cattle toward bovine brucellosis.

## 2. Materials and Methods

### 2.1. Study Areas

The study was conducted from November 2016 to April 2017 in the Loko and Uke peasant association of Guto Gida District, East Wollega, Oromia, Ethiopia. The altitude of the Uke and Loko is between 1700 and 1800 m a.s.l. and temperature is 26°C. It gets annual rainfalls of 1400 mm (Guto Gida fish and livestock office, 2009). Out of the twenty peasants' associations found in Guto Gida district, Loko and Uke are mostly known by their livestock population. Out of 122,364 cattle populations found in the Guto Gida district, 25000 (20.4%) were found in those selected PAs.

They are at 32 km, away from Nekemte Town. Mixed crop and livestock farming system is the mode of agriculture in the districts in which cattle are the dominant livestock, which is very important for the livelihood of the local population [28]. Study population: the study animals were indigenous cattle breeds kept under extensive management systems in the area. All cattle in the study area with the age of 6 months or above are part of the study.

### 2.2. Study Design, Sampling Method, and Sample Side

A cross-sectional study was conducted from November 2016 to April 2017 on free grazing Bovine Population found in Loko and Uke peasants associations of Guto Gida district, East Wollega zone, aiming to determine the prevalence of current bovine brucellosis. Animals were selected randomly using the lottery method. The sample size required for this study determined depends on the expected prevalence and the desired absolute precision.

The sample size was determined using the formula given in the trust field [29].

(1) $$\begin{array}{c} N=1.96^{2}\frac{p\left(1-p\right)}{D^{2}}=30, \end{array}$$

where *N* is the number of animals sampled, *p* is the expected prevalence of the disease, and *D* is the precision level (0.05). The expected prevalence of the disease was 1.97% [26]. Our calculated sample size was 30 animals, but to increase the precision, we inflated the sample size to about three times the original sample sizes of 80 animals [29].

### 2.3. Data Collection Methods

#### 2.3.1. Questionnaires Survey

The survey was conducted on two peasants' associations from November 2016 to April 2017. Two hundred (200) cattle owners were selected randomly and interviewed using a structured questionnaire. A detailed, organized, and structured questionnaire was prepared and administered in-person to farmers in an interview to get baseline information on the existing diseases of ruminants according to the farmers. The questionnaire was pretested in the field, adjusted as required, and translated into the local language that the farmers can understand.

#### 2.3.2. Blood Sample Collection

About 10 ml of blood volume was collected from the jugular vein of each selected cattle into a plain vacuum tube and allowed to clot in a slant position at room temperature, and the serum was decanted into a labeled vial and stored at −20°C until tested [30].

#### 2.3.3. Complement Fixation Test (CFT)

CFT was performed to test serum samples at the National Veterinary Institute (NVI), Debre Zeit, Ethiopia, according to the protocol described in [11]. Analysis data were stored in Microsoft Excel and transported to SPSS software version 20 for statistical analyses such as simple descriptive statistical analysis.

The seroprevalence is estimated by dividing the number of test-positive cattle by the total number of cattle tested. A chi-square test was applied to measure the association between the disease and risk factors such as sex, age, and site. In a 95% confidence interval, a *P* value of less than 0.05 is considered a statistically significant difference.

(2) $$\begin{array}{c} \text{Prevalence}=\frac{\text{total number of positive sample}}{\text{total number of sample}}\times 100. \end{array}$$

## 3. Results

### 3.1. Serological Results

Out of eighty cattle tested for bovine brucellosis, 22.5% (18/80) were positive for bovine brucellosis. The higher prevalence, 28.6%, was recorded at the Uke peasant association from the Guto Gida district, whereas the lower prevalence, 21.2% (14/66), was recorded at Loko (Table 1). Their differences in prevalence in two different sites were statistically insignificant (*P* > 0.05) (Table 1).

Out of eighty cattle tested for bovine brucellosis, 30.4% of males and 19.3% of females were positive for bovine brucellosis. The higher prevalence, 24.3%, was recorded in adults than young, but both sex and age were statistically insignificant (*P* > 0.05) (Table 2).

Statistically, there is an insignificant difference (*P* < 0.05) in *Brucella* antibodies for cattle in Uke peasant association when compared to those in Loko within the same district area from East Wollega, Oromia, Ethiopia.

### 3.2. Results of Questionnaire Survey

Out of 200 respondents, 53 (26.5%) were female, while 47 (73.5%) are male. Out of 200 respondents, 32 (16%) female and 64 (32%) male respondents have confirmed the occurrence of abortion on their farm. Only 2 (1%) females and 11 (5.5%) males believe that brucellosis was found in an urban area, while 51 (25.5%) females and 136 (68%) males believe that the existence of brucellosis was in the rural area (Table 3). Out of the two hundred respondents, 39 (19.5%) were illiterate, and 161 (80.5%) were literate. 21 (10.5%) illiterate respondents and 75 (37.5%) literate respondents have confirmed the existence of a history of abortion on their farm. 149 (74.5%) literate respondents believed that brucellosis has been highly found in urban area. Table 3 presents distribution of selected risk factors on general characteristics related to *Brucella* from the farmers.

On the other side, 38 (19%) illiterate respondents believed that this disease has been highly prevailed in a rural area (Table 3). About 29 (14.5%) females and 87 (43.5%) males were familiar with abortion occurring in the third trimesters, while 145 (72.5%) literate and illiterate respondents are well known for the occurrence of abortion at the third trimesters. These show that even multiple causative agents are present for abortion occurrence in the third trimester. However, it is a common clinical sign for brucellosis suspection (Table 4).

About 32 (16%) female and 128 (64%) male respondents believe in transmission brucellosis through consumption raw meat, milk, and contact fluid discharge. 156 (78%) age group respondents and 160 (80%) sex group respondents confirmed the transmission of disease from consumption and contact but did not know the specific disease (Table 4). Most of the female (16.5%) and male (54%) farmers followed traditional practice to expel out the retained placenta. However, female farmers (16.5%) prefer veterinarian compared to male farmers (7.5%) to expel out retained fetal membranes (Table 5).

Most of the farmers followed extensive management systems; relatively very few literate farmers are practicing intensive management systems. However, in using artificial insemination for breeding, there was a significant variation between illiterate and literate farmers, about 5% and 31.5%, respectively (Table 5). A comparison of farmers among age groups shows both young and adult prefer extensive management systems. However, regarding artificial insemination technology usage for breeding system, young farmers implemented it more than adult farmers, 20.5% and 16%, respectively (Table 5). The traditional skilled person was highly preferable in both literate and illiterate respondent farmers; however, several literate farmers practicing removal of retained fetal membrane by themselves were three wise lower than illiterate respondent farmers (Table 5).

124 (62%) respondents confirm mortality because of abortion in their herd annually: minimum was one and the maximum was five. 27 (13.5%) respondents confirm that over five abortion cases occurred annually. All respondents were well familiar with the economic importance of the disease in the country except 3 (1.5%) male respondents (Table 6).

## 4. Discussion

The overall prevalence of the present study findings, 22.5%, is lower than that, 38.7%, in the previous finding [31] in Bako Institute of Agricultural Research Farm and is closely related in agreement to 22% and 18.4% in Chafa State Dairy Farm and dairy farms around Addis Ababa, respectively [18, 32]. Our findings are higher than 16.65%, 8.2%, 8.11%, and 7.62% in and around Bahir Dar in North-Western Amhara and urban and periurban areas around Addis Ababa and Arsi region [21, 33, 34]. On the contrary, the present findings are extremely higher than those in the previous recorded reports by [35] with 2.1% in the Shoa region. By [26] with 1.97% in the Guto Gida district of East Wollega and by [36] with a prevalence of 1.92% in Sidama zone, other researchers also reported 0.05%, 0.77%, 0.45%, and 0.14% in the Arsi zone of Oromia regional state, Jimma zone, central highland, and Northern Gonder, respectively [37–40].

The higher overall prevalence of the present finding compared with previous extremely lower prevalence reports in the study area was for different justification. The first reason was to study animal sampling difference in the geographical location of Loko and Uke peasant association found in a lowland area of the district which is highly suitable for livestock production, especially for beef animals, and they are on the border of the district with the presence of fattening animals market.

Because of this reason, there is high livestock movement in and out of this area which may predispose factor for the disease. About 108 (54%) male respondents follow extensive management system; 128 (64%) respondents choose service of a farmer or unskilled personnel service for the expulsion of retained fetal membrane; this free movement may exaggerate the dissemination of the bacteria and source of infection. About 29 (14.5%) female respondents and 87 (43.5%) of male respondent were familiar with the occurrence of abortion in the third trimester; in addition, abortion occurrence in the third trimester was well known among 145 (72%) literate and illiterate respondents.

This shows that the third trimester was the pathognomonic symptom of brucellosis. This increased the devastating disease through horizontal transmission, and the event increased our findings. The second reason for the increment of finding is for the good sampling technique followed; in most of the previous study reports, their sampling sources were facility-based which were limited to representing the livestock population of the area, while our sampling framework included the whole cattle above six months of age found in the PAs without boundaries.

The third and final reasons were that most of the previously conducted studies used Rose Bengal Test for screening absent in our method; this also may play a crucial role in the increment of our findings for the good sensitivity of our diagnostic tool used. Sex has been one of the risk factors affecting the susceptibility of cattle to *Brucella abortus* infection [41]. In the present study, the prevalence of the disease among sex was 19.3% in females and 30.4% in males. The present findings are in contrary to different authors' findings of report on sex susceptibility to brucellosis by [33] around Addis Ababa [38] in the Jimma zone [42]. Because of this study, sex is not a risk factor for bovine brucellosis (*P* > 0.05).

Even though statistically significant variation among sex was not recorded, we recorded the higher seroprevalence in males than females; this may result in lack of awareness on the advantage of artificial insemination service. A large group of the respondents, about 128 (56%) literate male respondents, prefer natural mating, breeding system more than artificial insemination breeding system; therefore, during copulation, single infected bull may become a cause for infection of multiple bulls developed in single cow's mating; 90 (45%) male respondents used traditional skill man to remove the retained fetal membrane. This shows that devastating the disease through horizontal transmission for both humans and animals increased our finding.

Age is a risk factor in a previous study reported [c, 33, 43–46] because age is one of the intrinsic factors which can influence the susceptibility of *B. abortus* infection. In sexually mature animals, the predilection site was being the reproductive tract, especially the gravid uterus. This may be the factor that sex hormones and erythritol sugars which are found in the uterus that stimulate the growth and multiplication of *Brucella* organisms increase in concentration with age and sexual maturity.

However, sexually immature animals of either sex because of younger animals are more resistant to infection, frequently clear infections, and latent infections [46] However, in this study, age is a negative risk factor because about 93% of the age group were adult and only 6% of them were young; this low sample size may affect the association between which is statistically insignificant (*P* > 0.05). Conclusion and recommendations: bovine brucellosis is a highly prevalent disease in the study area. It is a probable cause of abortion and reproductive system problems in animals and humans.

Community knowledge, attitude, and practice level toward bovine brucellosis were poor. Therefore, based on the above conclusion, the following recommendations were forwarded.

Further detailed epidemiological studies are needed to investigate the link between bovine and human brucellosis. Strategic control measures should be formulated to reduce associated reproductive wastage and public health risk. For the continued rise of public awareness of brucellosis, it is necessary to raise the level of health knowledge and management practices.

Control measures need to be designed and implemented aiming at preventing further spread of the disease, like quarantine. Further investigation using reliable tools like the molecular technique are needed to know the exact epidemiological distribution of species of *Brucella*. To control the transmission of bovine brucellosis disease, strengthening the National Animal Health and Development Law should be layout by the government.

## Figures and Tables

**Table 1:** Seroprevalence of bovine brucellosis in two peasant association.

Variables	Category	Tested animals	Positive animals	Prevalence (%)	Chi-square	*P* value
Peasant association	Uke	14	4	28.6	0.035	0.00
	Loko	66	14	21.2		
	Total	80	18	22.5		

**Table 2:** Seroprevalence of brucellosis analyzed against age and sex.

Variables	Category	Tested animals	Positive animals	Percentage (%)
Sex	Male	23	7	30.4
	Female	57	11	19.3

Age	Young	6	0	0
	Adult	74	18	24.3

**Table 3:** Demography of respondent involved in questionnaire survey.

Variable	Category	Respondent	Percentage (%)
Education level of owner	Illiterate	39	19.5
	Primary	91	45.5
	Secondary	69	34.5
	above	1	0.5

Owner sex	Male	147	73.5
	Female	53	26.5

**Age (year)**	<30>15	38	19
	<45>31	64	32
	<60>46	76	38
	>60	22	11

Type of breed they own	Cross breed	22	11
	Local breed	176	88
	Unknown	2	1

**Table 4:** Distribution of selected risk factors on health care.

Factors affecting health care	Categories	Respondents	Percentage (%)
Management system	Extensive	119	59.5
	Intensive	17	8.5
	Semi-intensive	64	32

Breeding technique	Artificial insemination	55	27.5
	Natural	112	56
	Both	33	16.5

Vaccination against *Brucella*	No	186	93
	Yes	14	7

Way of eradication of *Brucella*	Culling of aborted animals		
	Yes	136	68
	No	64	32
	Control		
	Yes	134	67
	No	66	33

Where brucellosis highly found	Rural	16 9	84.5
	Urban	13	6.5
	Per urban	17	8.5
	None	1	½

**Table 5:** Distribution of selected risk factors on economic importance.

Economic important factors	Categories	Respondents	Percentage (%)
Term of abortion (Gatachiisa)	No	140	70
	Yes	60	30

Rate of abortion	1–5	124	62
	5–10	26	13
	>10	1	½
	None	49	24.5

Cause of abortion	Yes	96	48
	No	104	52

Loses of economy of country	Yes	197	98.5
	No	3	1.5

Period at animals aborted	First trimmers	37	18.5
	Second trimmers	7	3.5
	Third trimmers	115	57.5
	Unknown	41	20.5

**Table 6:** Distribution of selected risk factors on public health importance.

Factors	Categories	Respondents	Percentage
Transmission	Consumption	19	9.5
	Contact	37	18.5
	Through inhalation	2	1
	Both contact and consumption	142	71

Host affected by *Brucella*	Human being	92	31.5
	Large animals	103	35
	Small animals	6	2
	Avian	0	0
	All species' except avian	91	31

Whose acquired *Brucella* highly from the animals	Veterinarian	179	89.5
	Teachers	5	2.5
	Unknown	15	7.5
	None	1	½

## Data Availability

Data are available upon request to the first authors.
